# Nontesticular cancers in relatives of testicular germ cell tumor (TGCT) patients from multiple-case TGCT families

**DOI:** 10.1002/cam4.450

**Published:** 2015-04-17

**Authors:** Mary L McMaster, Ketil R Heimdal, Jennifer T Loud, Janet S Bracci, Philip S Rosenberg, Mark H Greene

**Affiliations:** 1 Division of Cancer Epidemiology and Genetics, National Cancer Institute, National Institutes of HealthBethesda, Maryland, 20892-9769; 2 Commissioned Corps of the U.S. Public Health Service, U.S. Department of Health and Human ServicesWashington, District of Columbia; 3 Section for Clinical Genetics, Department of Medical Genetics, Oslo University Hospital RikshospitaletOslo, Norway; 4 WestatRockville, Maryland, 20850-3157

**Keywords:** Familial cancer syndromes, genetic susceptibility, testicular germ cell tumors

## Abstract

Testicular germ cell tumors (TGCT) exhibit striking familial aggregation that remains incompletely explained. To improve the phenotypic definition of familial TGCT (FTGCT), we studied an international cohort of multiple-case TGCT families to determine whether first-degree relatives of FTGCT cases are at increased risk of other types of cancer. We identified 1041 first-degree relatives of TGCT cases in 66 multiple-case TGCT families from Norway and 64 from the United States (combined follow-up of 31,556 person-years). We collected data on all cancers (except nonmelanoma skin cancers) reported by the family informant in these relatives, and we attempted to verify all reported cancer diagnoses through medical or cancer registry records. We calculated observed-to-expected (O/E) standardized incidence ratios, together with 95% confidence intervals (CI), for invasive cancers other than TGCT. We found no increase in risk of cancer overall (Norway O/E = 0.8; 95% CI: 0.6–1.1 and United States O/E = 0.9; 95% CI: 0.7–1.3). Site-specific analyses pooled across the two countries revealed a leukemia excess (O/E = 6.5; 95% CI: 3.0–12.3), deficit of female breast cancer (O/E = 0.0; 95% CI: 0.0–0.6) and increased risk of soft tissue sarcoma (O/E = 7.2; 95% CI: 2.0–18.4); in all instances, these results were based on small case numbers and statistically significant only in Norway. While limited by sample size and potential issues relating to completeness of cancer reporting, this study in multiple-case TGCT families does not support the hypothesis that cancers other than testis cancer contribute to the FTGCT phenotype.

## Introduction

Although only 7920 incident cases of testicular germ cell tumor (TGCT) are expected in the United States this year 1, TGCT has substantial public health impact as the most common cancer affecting men aged 20–35 years. Several TGCT risk factors have been confirmed, including family history 2, previous contralateral testicular cancer 3, cryptorchidism 4, infertility 5, testicular microlithiasis 6, testicular developmental anomalies and atrophy 7,8, adult height 9, and in utero exposure to diethylstilbestrol 10. Apart from the contribution of these factors to TGCT development, this disease is notable for its striking heritability. Compared with most familial relative risks of 1.5- to 2.5-fold for first-degree relatives of common adult cancers, the risks to an individual whose brother or father has TGCT have been estimated as eight- to 10-fold and four- to sixfold, respectively 11–14, and the risk is further magnified in monozygotic twins 4. In addition, the Swedish Family Cancer database estimated that 25% of TGCT susceptibility can be attributed to genetic effects 15. Notwithstanding the pronounced heritability, large multigeneration TGCT families are rare and most families consist of relative pairs, most commonly brothers. The preponderance of sibling pairs suggests an autosomal recessive mode of inheritance, which is consistent with the model proposed by segregation analysis 16. However, multiple patterns of affection consistent with other modes of inheritance are regularly observed, including autosomal dominant (e.g., father and son) and X-linked (transmission through a female relative) inheritance. This suggestion of genetic heterogeneity has important ramifications for efforts devoted to identifying specific susceptibility genes.

Although initially promising 17, whole genome linkage analysis has not been successful in identifying highly penetrant TGCT susceptibility genes. Rather, overall linkage results suggested that susceptibility may be due to the combined action of multiple genes with smaller effects 18. Although candidate gene studies suggested several promising leads regarding the identity of these genetic modifiers 19–22, the current focus of TGCT gene discovery is driven by genome wide association studies (GWAS). To date, this research strategy has identified 18 genomic regions that are strongly associated with the risk of both sporadic and familial TGCT (FTGCT), and that have implicated multiple specific biological pathways in TGCT pathogenesis, for example, fertility, spermatogenesis, sex determination, testicular differentiation, double-stranded DNA break repair, spindle assembly checkpoint proteins, chromosomal segregation, chromatin remodeling, telomere maintenance, and apoptosis 23–27. While variation at some of these genomic regions confers unusually strong effects on risk, they do not account for all the predicted heritability in this disease.

In the search for genetic determinants of complex disease, refinement of the phenotype increases the statistical power to identify disease-related genes 28. With advances in high-throughput genetic technologies, approaches that elicit additional phenotypic information and increase power in linkage studies will have similar impact upon GWAS and whole genome and exome sequencing studies 29. Hereditary kidney cancer represents a prototypic illustration of this phenomenon, in which refinement of syndrome-specific histological subtypes has been instrumental in gene discovery efforts 30. In several other familial cancer syndromes, cancers other than the primary malignancy of interest have been found to occur excessively in at-risk family members, such as hereditary breast-ovarian cancer, multiple endocrine neoplasia types I and II, and hereditary nonpolyposis colon cancer, among others. Several prior studies have analyzed cancer risk in relatives of nonfamilial (sporadic) TGCT patients 12,14,31–44, but an excess risk of testicular cancer has been the only consistent finding in these reports. Results related to cancers other than TGCT have been inconsistent and contradictory. The cosegregation of multiple cancer sites within high-risk pedigrees can significantly increase the statistical power of gene discovery and molecular genetic studies, as well as provide evidence on which to base screening and interventional strategies. Therefore, we undertook a study of an international cohort of multiple-case TGCT families to determine whether cancers other than testis cancer may be part of the FTGCT phenotype.

## Materials and Methods

### Study population

Families with at least two histopathologically confirmed cases of TGCT, or a combination of TGCT and extragonadal germ cell tumor, and with DNA from at least one affected case were enrolled. Norwegian and U.S. participants were identified through physician- and self-referral, and were enrolled in protocols approved by the ethical and Institutional Review Boards of the Norwegian Radium Hospital (Registration Number 2011/625) and the National Cancer Institute (NCI) Clinical Genetics Branch (NCI protocol 02-C-0178, NCT-00039598), respectively. Results from a similar evaluation of cancer risk among the relatives of Norwegian TGCT probands have been previously published, and showed no significant cancer excesses 32. Ten percent of this cohort had familial/bilateral TGCT, but the number of cancers among relatives was deemed too small for informative analysis. We decided to reevaluate the Norwegian families in light of additional follow-up data and the availability of the comparable NCI cohort. While the Norwegian cohort is entirely referral-based, diagnoses could be confirmed using either pathology records or records from the Norwegian Cancer Registry. The NCI cohort comprises patients referred from multiple different providers throughout the United States. Although there were no exclusions based on race, all participating families were self-reported as white; data regarding ethnicity were not routinely collected. Participants provided informed consent for use of their deidentified demographic data and family history information, as well as consent to obtain medical records to validate diagnoses of cancers that were included in this analysis. Eligibility and clinical and demographic data were ascertained by the enrolling centers. Data for deceased family members were reported by study participants or obtained from their next-of-kin. Further information regarding the design and methods related to the NCI Clinical Genetics Branch cohort has been previously published 21,22,24.

## Data collection

In a given family, the first TGCT patient referred to the enrolling center was designated the index TGCT case and served as the initial informant for the family. At enrollment, we obtained from the informant information regarding family structure and a retrospective history of all cancers reported to have occurred in any family members. All cancers were classified according to ICD10 and/or ICD-O-3 criteria. For each reported cancer, we obtained independent confirmation of the diagnosis whenever possible, including review of medical and pathology records, death certificates and national cancer registry data (the latter in Norway only). For all family members, we recorded gender, vital status, genetic relationship to the index TGCT case, date of birth, and dates of death and cancer diagnosis, as applicable. Data collected at the family level included number, distribution and type (seminoma or nonseminoma) of TGCT cases, apparent mode of inheritance of TGCT, and history of undescended testis or congenital hernia. The relationship between cases in each family was classified as siblings, first cousins, father–son, uncle–nephew, and complex. The complex category included families in which the relationship between the cases did not fit one of the specific categories, or consisted of a combination of two or more of those categories (e.g., a family with two maternal first cousins and their mothers’ brother contained one first cousin and two uncle–nephew relationships). All family members were categorized in two different ways based on the genetic distance (e.g., first-degree) and character (e.g., sibling) of their relationship: (1) to the index TGCT case, and (2) to the most closely genetically related TGCT case. For example, in a family containing two first cousins with TGCT, the siblings of the nonindex case would be classified as third-degree first cousins of the index case and first-degree siblings of the nearest case. Pedigrees were inspected for apparent mode of inheritance and categorized as being definitely compatible (Yes) or definitely incompatible (No), with each of three major inheritance patterns: autosomal dominant, autosomal recessive, and X-linked. If a mode of inheritance could not be excluded, the family was coded as “Possible.” For example, a father–son pair would be categorized as autosomal dominant-Yes, autosomal recessive-Possible, and X-linked recessive-No.

Referent age-adjusted population cancer incidence rates were compiled, stratified by gender, 5-year age groups and 5-year calendar periods using data from the Cancer Registry of Norway (1953–2000) and the National Cancer Institute’s Surveillance, Epidemiology and End-Results (SEER) nine-registry database (1973–2008) (SEER Program Research Data, 1973–2008; www.seer.cancer.gov; 2010 submission). Using the assigned ICD10 (Norway) and ICD-O-3 (United States) codes, referent cancers were grouped according to the SEER 9 Site Recode variable (Site Recode ICD-O-3 (1/27/2003); http://seer.cancer.gov/siterecode/index.html). Cancers of unknown primary site were classified as Miscellaneous.

## Statistical analysis

We used the collected data to construct an analytic cohort consisting of first-degree relatives of TGCT cases. We excluded individuals who did not share the bloodline of all TGCT cases in the family (*n* = 879) or who had unknown vital status (*n* = 54) or missing dates of birth (*n* = 167) or death (*n* = 120) from the analysis. Because of variations in cancer incidence rates over time, we limited the analysis to cancers diagnosed during the periods for which referent cancer incidence rates were available (Norway, 1953–2000; United States, 1973–2008). For each individual, only the first invasive nontesticular germ cell cancer was included in the observed cancer counts. Noninvasive cancers and nonmelanoma skin cancers were excluded. If the date of cancer diagnosis was unknown, then the date was set to the study cut-off date of 31 December 2007.

Accrued person-years were calculated for the cohort, with study entry date defined as date of birth, and study exit date the earliest of (1) first invasive non-TGCT cancer, (2) death, or (3) study cut-off. We calculated the observed-to-expected (O/E) standardized incidence ratio (SIR) for invasive cancers other than TGCT occurring in this cohort using the Multiple Primary Standardized Incidence Ratios function of SEER*Stat (Surveillance Research Program, National Cancer Institute SEER*Stat software (seer.cancer.gov/Seerstat, version 8.1.2). This program provides a method to follow a defined cohort over time to compare their cancer diagnoses with the number of cancers that would be expected based on incidence rates for the general population. The expected number of cancers was calculated by applying general population cancer incidence rates specific to registry, age (in 5-year groups), and calendar year (in 5-year groups), to person-years accrued by cohort members. The SIRs were calculated overall and stratified by contributing center and gender but not adjusted for relationship between relatives other than cases. The SIRs for the combined registry data were calculated by combining the observed and expected case counts for each center. Exact Poisson 95% confidence intervals (95% CIs) were calculated for the SIR estimates 45. We considered 95% CIs that excluded 1.00 to be statistically significant. To assess whether any site-specific SIRs remained statistically significant after adjusting for multiple testing, we calculated False Discovery Rate (FDR) adjusted *P*-values 46 for Norway, the United States, and for the entire set of cases combined. We considered adjusted *P*-values less than or equal to 0.05 to be statistically significant. For some cancers, the Norwegian Cancer Registry and the SEER Program differed in number of subsites per cancer site for which population incidence rates were calculated. For our analyses, the least restrictive cancer site groupings were used. For example, we used “leukemia” as a combined grouping because incidence rates per subtype were not available for Norway.

## Results

A total of 1041 subjects from 130 families, with a combined follow-up of 31,556 person-years, were included in this analysis (Norway: 586 subjects from 66 families; United States: 455 subjects from 64 families). Table1 depicts the characteristics of the cohort. Overall the cohort was well-balanced between the two centers, with a few exceptions. At the family level, uncle–nephew TGCT pairs and families in which cases exhibited both seminoma and nonseminoma were over-represented among Norwegian families compared with those from the United States. At the individual level, subjects from the United States were more likely to be first-degree relatives of the index cases.

**Table 1:** Selected characteristics of familial testicular germ cell tumor (TGCT) cases and case families from Norway and the United States

Characteristic	Norway	United States	Combined
Family-level data			
Total number of multiple-case families, *n* (%)	66 (51)	64 (49)	130 (100)
No. of TGCT cases per family, *n* (%)			
≥3	8 (12)	13 (20)	21 (16)
2^1^	58 (88)	51 (80)	109 (84)
Relationship pattern between TGCT cases, *n* (%)			
Father–son	13 (20)	14 (22)	27 (21)
Siblings^2^	22 (33)	30 (47)	52 (40)
Cousins	9 (14)	9 (14)	18 (14)
Uncle–nephew^3^	13 (20)	1 (2)	14 (11)
Complex	9 (14)	10 (16)	19 (15)
TGCT histology within families, *n* (%)			
Seminoma only^3^	17 (26)	28 (44)	45 (35)
Nonseminoma only	8 (12)	15 (23)	23 (18)
Both seminoma & nonseminoma^3^	38 (58)	21 (33)	59 (45)
Unknown^4^	3 (5)	0 (0)	3 (2)
Apparent mode of inheritance,^5^ *n* (%)			
Autosomal dominant—yes	15 (23)	21 (33)	36 (30)
Autosomal recessive—yes	31 (47)	38 (59)	69 (57)
X-linked—yes	11 (17)	5 (8)	16 (13)
Subject-level data			
Total number of participants, *n* (%)	586 (56)	455 (44)	1041 (100)
Sex, *n* (%)			
Male	303 (52)	229 (50)	532 (51)
Female	283 (48)	226 (50)	509 (49)
Degree relationship to index TGCT case, *n* (%)			
1st degree^3^	326 (56)	310 (68)	636 (61)
2nd degree	167 (29)	108 (24)	275 (26)
≥3rd degree^3^	93 (16)	37 (8)	130 (13)
Type of relationship to nearest TGCT Case,^6^ n			
Parent	136	113	249
Sibling	294	180	474
Offspring	170	198	368

Percentages may not sum to 100 due to rounding.

1 Includes three U.S. families each with one case of extragonadal germ cell tumor.

2 Includes half-siblings (*n* = 1) and monozygous twins (*n* = 1).

3 Nominal *P *<* *0.05.

4 In each of three Norwegian families, histological type was unknown for one TGCT case.

5 See Materials and Methods for how these classifications were determined. For 14 families, pedigree data were too ambiguous to permit classification. These included eleven families from Norway and three families from the United States

6 Eighty subjects had a first-degree relationship to more than one TGCT case, for example, a single individual might be mother of one TGCT case and sister of a second TGCT case. Thus, these categories do not sum to total.

Eighty-four non-TGCT cancers were observed among first-degree relatives of the nearest TGCT case during the study period (Norway = 42; United States = 42). Of these, 73.8% were validated by pathology report or cancer registry data (Norway = 34, 81.0%; United States = 28, 66.7%). Validation proportions were similar for cancers in first-degree and second-degree relatives of index cases but dropped sharply (50.0%, *n* = 4) for more distantly related individuals.

No excess of all cancers combined was observed at either center (Table2). The O/E ratios were 0.8 (95% CI = 0.6–1.1) and 0.9 (95% CI = 0.7–1.3) in Norway and the United States, respectively. Specific site analyses revealed an excess of leukemia, all types combined, among subjects at both centers; however, this was statistically significant only for Norway, and was based on a limited number of cases (*n* = 6), leading to a wide CI (Norway; *n* = 6; O/E = 48.8; 95% CI = 17.9–106.2). Similarly, there was an excess of soft tissue sarcomas (Norway; *n* = 4; O/E = 14.5; 95% CI = 4.0–37.1) and a deficit of female breast cancer (Norway; *n* = 0; O/E = 0.0; 95% CI 0.0–0.6) that were significant only among Norwegian subjects. These associations remained significant after adjusting for multiple testing (FDR adjusted *P*-values <0.01). The breast cancer deficit remained significant in Norway at the 0.05 level after adjustment. However, no site-specific excess or deficit was statistically significant in the United States study, and only leukemia remained significant following adjustment for multiple testing in the combined data. We then performed a sensitivity analysis, using only independently validated cancers, which did not substantially alter the site-specific results (data not shown). A second analysis, restricted to first-degree relatives of the index TGCT cases, yielded similar results (Table3).

**Table 2:** Standardized incidence ratios and 95% CI for cancers other than testicular germ cell tumors (TGCT) occurring in first-degree male and female relatives of TGCT cases in multiple-case TGCT families from Norway and the United States

	Norway				United States				United States and Norway			
No. persons	586				455				1041			
Person-years at risk	19,748.5				11,807.2				31,555.7			
Cancer site	Obs	Exp	O/E	95% CI	Obs	Exp	O/E	95% CI	Obs	Exp	O/E	95% CI
All sites^1^	42	50.2	0.8	0.6–1.1	42	44.5	0.9	0.7–1.3	84	94.7	0.9	0.7–1.1
Oral cavity and pharynx	0	1.2	0.0	0.0–3.1	1	1.2	0.9	0.0–4.7	1	2.4	0.4	0.0–2.4
Digestive system	12	12.9	0.9	0.5–1.6	8	7.8	1.0	0.4–2.0	20	20.7	1.0	0.6–1.5
Stomach	3	3.0	1.0	0.2–2.9	1	0.7	1.5	0.0–8.4	4	3.7	1.1	0.3–2.8
Colon and rectum	8	7.1	1.1	0.5–2.2	5	4.8	1.0	0.3–2.4	13	11.9	1.1	0.5–1.9
Pancreas	1	1.6	0.6	0.0–3.6	2	1.0	2.0	0.3–7.3	3	2.6	1.2	0.2–3.4
Respiratory system	3	5.3	0.6	0.1–1.7	7	6.6	1.1	0.4–2.2	10	11.9	0.8	0.4–1.5
Lung and bronchus	3	4.6	0.7	0.1–1.9	6	6.1	1.0	0.4–2.2	9	10.7	0.8	0.4–1.6
Soft tissue^2^	**4**	**0.3**	**14.5**	**4.0–37.1**	0	0.3	0.0	0.0–13.1	4	0.6	7.2	2.0–18.4
Melanoma	3	2.4	1.3	0.3–3.7	1	2.0	0.5	0.0–2.8	4	4.3	0.9	0.3–2.4
Breast^3^	**0**	**6.5**	**0.0**	**0.0–0.6**	6	7.3	0.8	0.3–1.8	6	13.7	0.4	0.2–1.0
Female genital	3	5.1	0.5	0.1–1.7	0	3.2	0.0	0.0–1.2	3	8.3	0.4	0.1–1.1
Prostate	3	5.3	0.6	0.1–1.7	6	5.6	1.1	0.4–2.3	9	10.9	0.8	0.4–1.6
Urinary system	1	4.0	0.3	0.0–1.4	4	3.1	1.3	0.4–3.3	5	7.2	0.7	0.2–1.6
Thyroid	0	0.6	0.0	0.0–5.9	1	0.8	1.3	0.0–7.1	1	1.4	0.7	0.0–3.9
Lymphoma	2	1.5	1.3	0.2–4.8	4	2.2	1.9	0.5–4.7	6	3.7	1.6	0.6–3.6
Leukemia^4^	**6**	**0.1**	**48.8**	**17.9–106.2**	3	1.3	2.4	0.5–6.9	**9**	**1.4**	**6.5**	**3.0–12.3**

Statistically significant results following adjustment for multiple comparisons are shown in bold type. Obs, observed; Exp, expected; O/E, ratio of observed to expected; CI, confidence interval.

1 Major organ sites having at least one observed case are presented. Neither center observed cases of multiple myeloma or cancers affecting bone and joint, eye and orbit, or brain.

2 Soft tissue sarcomas included one each of neurofibrosarcoma, leiomyosarcoma, endometrial stromal sarcoma, and mixed mesenchymal sarcoma.

3 Breast cancer category includes only females for Norway and both genders for United States

4 Leukemias include: acute lymphocytic leukemia (*n* = 2), acute myelocytic leukemia (*n* = 1), chronic lymphocytic leukemia (*n* = 3), chronic myelogenous leukemia (*n* = 1), and leukemia, not otherwise specified (*n* = 2).

**Table 3:** Standardized incidence ratios and 95% CI for cancers other than testicular germ cell tumor (TGCT) occurring in first-degree male and female relatives of index TGCT cases in multiple-case TGCT families from Norway and the United States

	Norway				United States				United States and Norway			
No. persons	363				367				730			
Person-years at risk	12,535.6				10,334.5				22,870.1			
Cancer site	Obs	Exp	O/E	95% CI	Obs	Exp	O/E	95% CI	Obs	Exp	O/E	95% CI
All sites^1^	25	25.8	1.0	0.6–1.4	31	33.2	0.9	0.6–1.3	56	59.1	1.0	0.7–1.2
Oral cavity and pharynx	0	0.8	0.0	0.0–4.5	1	0.9	1.1	0.0–6.0	1	1.5	0.7	0.0–3.6
Digestive system	7	6.5	1.1	0.4–2.2	5	5.6	0.9	0.3–2.1	12	12.0	1.0	0.5–1.7
Stomach	1	1.5	0.7	0.0–3.8	0	0.5	0.0	0.0–7.7	1	2.0	0.5	0.0–2.8
Colon and rectum	5	3.6	1.4	0.5–3.3	4	3.4	1.2	0.3–3.1	9	6.9	1.3	0.6–2.5
Pancreas	1	0.8	1.3	0.0–7.1	1	0.7	1.5	0.0–8.1	2	1.5	1.4	0.2–4.9
Respiratory system	4	2.7	1.5	0.4–3.9	7	4.8	1.5	0.6–3.0	11	7.5	1.5	0.7–2.6
Lung and bronchus	4	2.3	1.7	0.5–4.4	6	4.4	1.4	0.5–3.0	10	6.7	1.5	0.7–2.7
Soft tissue^2^	**2**	**0.2**	**12.5**	**1.5–45.2**	0	0.2	0.0	0.0–16.0	2	0.4	5.1	0.6–18.3
Melanoma	1	1.4	0.7	0.0–4.1	2	1.7	1.2	0.2–4.6	3	3.0	1.0	0.2–2.9
Breast^3^	0	3.5	0.0	0.0–1.1	3	5.1	0.6	0.1–1.7	3	8.5	0.4	0.1–1.0
Female genital	1	2.7	0.4	0.0–2.0	0	2.2	0.0	0.0–1.7	1	4.9	0.2	0.0–1.1
Prostate	2	2.4	0.8	0.1–3.0	5	4.4	1.1	0.4–2.7	7	6.8	1.0	0.4–2.1
Urinary system	0	2.0	0.0	0.0–1.8	2	2.4	0.8	0.1–3.1	2	4.4	0.5	0.1–1.7
Thyroid	0	0.4	0.0	0.0–10.3	1	0.7	1.5	0.0–8.4	1	1.0	1.0	0.0–5.5
Lymphoma	0	0.9	0.0	0.0–4.3	1	1.8	0.6	0.0–3.2	1	2.6	0.4	0.0–2.1
Leukemia^4^	**5**	**0.1**	**83.3**	**22.1–194.5**	2	1.0	2.1	0.3–7.6	**7**	**1.0**	**6.9**	**2.8–14.3**

Statistically significant results (nominal *P*-values <0.05) are shown in bold type. Obs, observed; Exp, expected; O/E, ratio of observed to expected; CI, confidence interval.

1 Major organ sites having at least one observed case are presented. Neither center observed cases of multiple myeloma or cancers affecting bone and joint, eye and orbit, or brain.

2 Soft tissue sarcomas included one each of neurofibrosarcoma and leiomyosarcoma.

3 Breast cancer category includes only females for Norway and both genders for United States

4 Leukemias include: acute lymphocytic leukemia (*n* = 1), acute myelocytic leukemia (*n* = 1), chronic lymphocytic leukemia (*n* = 2), and leukemia, not otherwise specified (*n* = 2).

We were also interested in whether specific characteristics that differed between families might influence cancer patterns within families. We therefore performed a series of analyses stratified by variables of interest, including the number of TGCT cases in a family (2 vs. ≥3), histological type of TGCT segregating within families (seminoma only vs. nonseminoma only vs. both seminoma and nonseminoma), pattern of relationship between TGCT cases within families (siblings vs. father and son pairs vs. uncle and nephew pairs), apparent mode of inheritance (autosomal dominant vs. autosomal recessive vs. X-linked recessive), and the type of relationship of the relative to the nearest TGCT case (siblings vs. parents). In all these subanalyses, we saw no substantial difference in the results compared with the cohort overall (Table4). In this set of analyses, we limited the site-specific analyses to those sites that had shown excesses or deficits in the main analysis. Although numbers in individual cells were small, the data suggested possible differences in risk in various subgroups. For example, it appeared that families with X-linked recessive inheritance or uncle and nephew pairs might have higher excess risks for leukemia and soft tissue tumors. When we examined individual cases of leukemia and soft tissue tumors, however, we found that each cancer site contained a variety of histological subtypes, with no consistent pattern observed.

**Table 4:** Standardized incidence ratios and 95% CI for specific cancers other than testicular germ cell tumors (TGCT) occurring in first-degree male and female relatives of TGCT cases in Norway and the United States stratified by TGCT case or case family characteristics

Stratification characteristic	PY	Overall			Soft tissue			Breast			Leukemia		
		Obs	O/E	95% CI	Obs	O/E	95% CI	Obs	O/E	95% CI	Obs	O/E	95% CI
No. of TGCT cases in family													
2	24,911	63	0.9	0.7–1.1	**3**	**7.0**	**1.4–20.4**	**4**	**0.4**	**0.1–1.0**	**8**	**7.7**	**3.3–15.2**
≥3	6641	21	1.0	0.6–1.6	1	7.7	0.2–42.9	2	0.6	0.1–2.3	1	2.9	0.1–15.9
Histological type of TGCT in family													
Seminoma only	9447	32	1.1	0.7–1.5	**2**	**11.8**	**1.4–42.5**	3	0.7	0.0–3.9	2	3.6	0.4–12.9
Nonseminoma only	5587	13	0.9	0.5–1.5	0	0.0	0.0–36.9	1	0.4	0.0–2.4	**3**	**9.7**	**2.0–28.3**
Both seminoma and nonseminoma	15,626	37	0.8	0.6–1.1	2	7.4	0.9–26.8	2	0.3	0.0–1.1	**4**	**7.8**	**2.1–20.1**
Pattern of TGCT in family													
Siblings	10,109	25	0.9	0.5–1.4	1	5.9	0.2–32.8	1	0.2	0.0–1.3	1	2	0.1–11.1
Father/son	4508	20	1.0	0.6–1.5	0	0.0	0.0–36.9	1	0.4	0.0–2.0	2	5.6	0.7–20.1
Uncle/nephew	7479	22	0.9	0.6–1.4	**3**	**23.1**	**4.8–67.4**	0	0.0	0.0–1.2	**5**	**50.0**	**16.2–116.7**
Inheritance model													
Autosomal dominant	6940	28	1.0	0.6–1.4	0	0.0	—	3	0.7	0.2–2.1	2	3.5	0.4–12.5
Autosomal recessive	14,874	28	0.8	0.5–1.1	1	4.2	0.1–23.2	3	0.5	0.1–1.6	1	1.7	0.0–9.4
X-linked recessive	6667	15	0.9	0.5–1.4	**2**	**20**	**2.4–72.3**	2	0.8	0.1–2.8	**4**	**30.8**	**8.4–78.8**
Unclear inheritance	4439	15	1.1	0.6–1.9	1	12.5	0.3–69.7	0	0.0	—	**2**	**22.2**	**2.7–80.3**
Type of relation to nearest TGCT case													
Siblings	17,634	33	0.9	0.6–1.2	2	8.0	1.0–28.9	4	0.7	0.2–1.7	**4**	**8.9**	**2.4–22.8**
Parents	8409	51	0.9	0.7–1.2	2	7.7	0.9–27.8	**2**	**0.3**	**0.0–0.9**	**4**	**4.7**	**1.3–12.1**

Statistically significant results (nominal *P*-value <0.05) are shown in bold type. PY, person-years at risk; Obs, number observed; O/E, ratio of observed to expected; CI, confidence interval.

## Discussion

In this quantitative study of cancer risk within multiple-case TGCT families, we found no excess risk of non-TGCT cancers at all sites combined among first-degree relatives of TGCT cases. While there was limited evidence supporting altered site-specific risks for soft tissue tumors, leukemias and breast cancer, our results were not consistent, either relative to the literature or between the two cohorts, suggesting that differences in cancer reporting among families, case ascertainment, validation methods and success, or other factors, rather than an etiologic association, may explain these findings. It is possible that a subset of families have risks for other cancers that cannot be determined because of limited power and extensive heterogeneity. Until larger FTGCT cohorts are available and more robust parameters are identified to better classify families, however, our data suggest that FTGCT may be a cancer site-specific syndrome. In general, our current understanding of TGCT genetics supports this interpretation in that most identified variants are of low penetrance, and many (though not all) are located in or near genes known to influence primordial germ cell maturation and differentiation and are therefore less likely to be associated with other cancer types. If confirmed, there are two major consequences of this observation: (1) we will not be able to leverage a broader syndromic phenotype—had one been found—with increased statistical power toward improved FTGCT gene discovery efforts; and (2) the available evidence does not suggest that the clinical management of FTGCT kindred should be broadened to include surveillance for, and risk-reducing strategies aimed at, cancers other than TGCT itself.

We observed increased risks of leukemia (all subtypes combined) and soft tissue cancer (multiple histological subtypes combined) in our study that persisted throughout most stratified analyses and following adjustment for multiple testing. The highest risks for both were observed in families with affected uncle–nephew TGCT pairs, a pattern compatible with an X-linked recessive mode of inheritance. One of the seminal FTGCT gene discovery linkage analyses implicated an X-chromosome susceptibility locus 17, but the evidence supporting genetic linkage at that locus diminished substantially when a much larger number of families was analyzed 18. Interestingly, leukemias and sarcomas are among the most common second malignancies observed in TGCT patients following successful therapy for their testicular cancer, but these associations appear to be mainly due to late carcinogenic effects of treatment, rather than shared etiologic factors 47. Our findings were strongest for leukemia risk, which was elevated when all subtypes were considered in the aggregate in both centers, but statistically significant only in the Norwegian families. However, among the seven leukemia cases having more detailed information available, there was heterogeneity in both cell lineage (five lymphoid and two myeloid) and differentiation state (three acute, four chronic). In general, these various histological leukemia subtypes are regarded as etiologically distinct disorders, so it is not entirely logical to analyze them as a group in a study such as this one. For this to make biological sense, one would need to argue that any shared genetic abnormality or critical environmental exposure must occur at a very early stage in hematopoietic differentiation to produce such diverse outcomes. The data to support such an hypothesis are sparse. Prior investigators have differed in their approach to classifying leukemias and lymphoid cancers by considering them either together or separately. None, however, has provided details about the subtype classification of observed leukemias, including four studies that found small nonsignificant 33,43,48 to moderate significant 41 elevations in hematopoietic cancers in subsets of sporadic TGCT relatives.

Soft tissue sarcomas, derived from mesenchymal tissue, are typically classified according to their eventual differentiation pattern as determined by morphology and immunohistochemistry. Sarcoma classification continues to evolve, influenced by results of genomic and gene expression studies, which indicate that sarcomas can be further characterized by the complexity of their genomic alterations 49. Although overrepresented as a group, the soft tissue sarcomas observed in this study were heterogeneous in subtype, with four different morphologic subtypes represented. The single other study that reported incidence of soft tissue sarcomas in relatives of testis cancer patients 38 observed no increase in bone and soft tissue tumors in first-degree relatives of patients with testis cancer; this category of tumors has not been reported in other similar investigations. Thus, these two observations must be interpreted with caution in light of the small numbers of cases. If they were to be confirmed, they suggest the possibility that leukemia and soft tissue sarcomas might share etiological factors with testicular cancer. At present, our data do not warrant drawing such a conclusion. We believe it is crucial that future studies be adequately powered to separate these broad diagnostic categories into biologically distinctive subtypes. Given the rarity of TGCT families and the time- and resource-intensive nature of TGCT family ascertainment and evaluation, one potential mechanism for accomplishing this goal might be to pool data from this and previous studies to perform a meta-analysis. Alternatively, a carefully coordinated consortial approach might permit the accumulation of adequate numbers to address these questions in a definitive manner.

The extent to which familial clustering of TGCT is due to shared genetic or environmental exposures is unclear. An early report of elevated breast cancer risk among first-degree female relatives of sporadic TGCT cases 50 led to the hypotheses that common prenatal exposures or relative estrogen excess might have a role in susceptibility to cancer arising in hormonally sensitive tissues in both mothers and siblings of TGCT patients. In the original Norwegian study 32, a decrease in prostate cancer risk in fathers of TGCT patients was observed. Subsequent studies that examined hormone-dependent cancers have shown the strongest effects for breast cancer 13,35,39,43. However, the breast and prostate cancer results have not been replicated consistently, either historically 34,37,41,42 or in this study. Instead, we found a decreased risk of breast cancer in our familial cohort that appeared to be restricted to mothers of cases, but this was based on only two cases and was marginally significant in only one center. Moreover, we found no significant change in prostate cancer risk for relatives of TGCT patients compared to that expected in the general population in either center. These differences between studies could result from a variety of factors related to study design or pathobiology (e.g., genetic heterogeneity, phenotypic pleiotropy, or the influence of unidentified environmental factors). A Danish analysis 33 that found a reduced breast cancer risk among mothers of TGCT patients hypothesized that the reduction was related to the relative protective effect of parity. We did not have information on parity or other maternal risk-related factors, and thus were unable to test this notion in our cohort. One breast cancer was observed in the Norwegian cohort but was excluded from the analysis due to critical missing data (i.e., missing date of diagnosis). Thus, the apparent significant deficit of breast cancer may be an artifact related to incomplete data.

Our analysis was limited by sample size, differences in case ascertainment between centers, retrospective cancer reporting, and incomplete data. In addition, while we attempted to validate the cancer diagnosis in those relatives reported to have cancer, we did not systematically confirm the absence of cancer in those reported to be disease-free. However, the literature suggests that reports of “no cancer” are much more accurate than reports of specific cancers 51, thus mitigating this potential shortcoming. Furthermore, our results may not be generalizable to other FTGCT cohorts.

The strengths of this analysis include: (1) the evaluation of two geographically distinct cohorts using the same methodology; (2) the collection of the largest set of multiple-case TGCT families ever assembled to address this research question; (3) the systematic effort employed to document reported cancer diagnoses, a critical step given the general unreliability of reported cancer histories in family studies 51; (4) the formal quantification of cancer risks using country-specific, population-based, cancer site-specific incidence rates; and (5) the availability of extensive covariate data, permitting exploration of possible associations in prespecified subgroups of interest.

In summary, limited data suggest some potentially interesting alterations in site-specific risks in TGCT families that deserve further evaluation in larger studies. Leukemias and soft tissue cancers were statistically significantly more common in the Norwegian families with TGCT when multiple histological subtypes were combined for each site. However, these associations were not found among the U.S. families. Clearly, there is a continuing need for large, well-designed studies to address issues of phenotypic and genetic heterogeneity in this disease.
